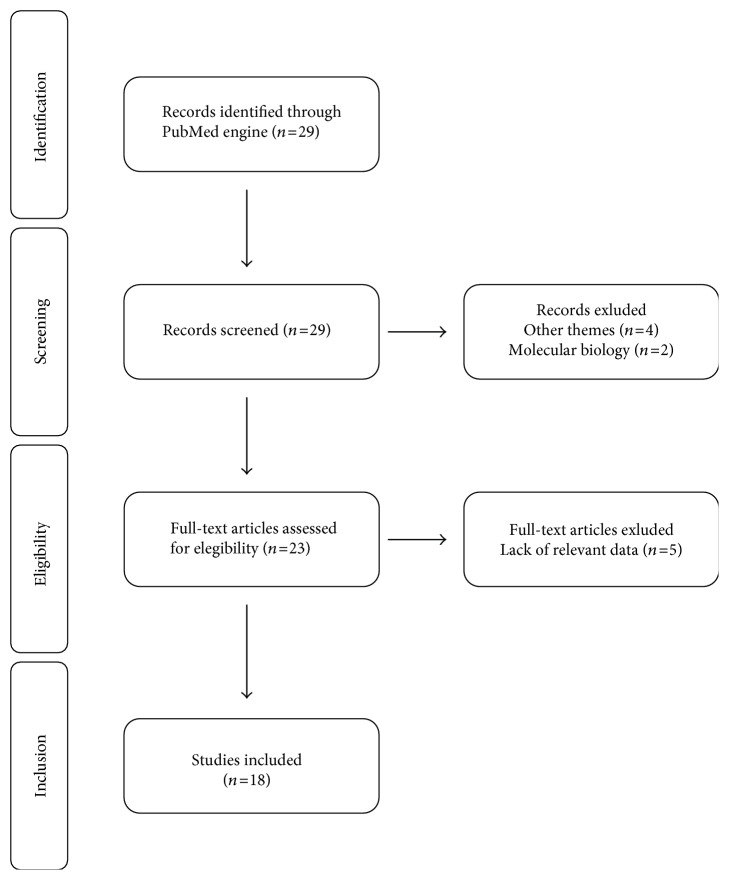# Immunohistochemical Markers as Predictors of Histopathologic Response and Prognosis in Rectal Cancer Treated with Preoperative Adjuvant Therapy: State of the Art

**DOI:** 10.1155/2017/2808235

**Published:** 2017-02-23

**Authors:** Alessandro Del Gobbo, Stefano Ferrero

**Affiliations:** ^1^Division of Pathology, Fondazione IRCCS Ca' Granda Ospedale Maggiore Policlinico, University of Milan, Via Francesco Sforza 25, 20122 Milan, Italy; ^2^Department of Biomedical, Surgical and Dental Sciences, University of Milan Medical School, Via Francesco Sforza 35, 20122 Milan, Italy

## Abstract

We explain the state of the art of the immunohistochemical markers of response in rectal cancers treated with neoadjuvant medical therapies and its implication with prognosis. Neoadjuvant chemoradiotherapy is widely used to improve the outcome of patients with locally advanced rectal cancer, and the evaluation of the effects of medical therapy is to date based on histomorphological examination by applying four grading systems of response to therapy (tumor regression grade (TRG)). The need to identify immunohistochemical markers that could ensure a better assessment of response and possibly provide additional prognostic information has emerged. We identified p53, p27kip1, Ki67, matrix metalloprotease-9, survivin, Ki67 proliferative index, CD133, COX2, CD44v6, thymidylate synthase, thymidine phosphorylase, and dihydropyrimidine dehydrogenase as the most common markers studied in literature to date, and we explained their prognostic potential and their implications in the evaluation of the response to preoperative therapies in rectal cancers.

## 1. Introduction

Neoadjuvant chemoradiotherapy is a useful tool to improve outcome of patients with locally advanced rectal cancer, in terms of both local recurrence and overall survival, in particular in rectal adenocarcinomas.

After surgery, the evaluation of the effects of medical therapy is usually based on histopathological examination of the surgical specimen.

Four grading systems of response to therapy (tumor regression grade (TRG)) related to prognosis are normally used by pathologists to date, and they are elaborated by Becker, Mandard, Dworak, and Rödel.

These grading systems are usually based on the percentage of residual tumor cells in relation to fibrotic reaction to neoadjuvant treatments; some of them were elaborated for other organs of the gastrointestinal district and two of them (Dworak and Rödel) are applied also in rectal cancers [1–4].

Alongside pure morphological histomorphological examination, a need to identify immunohistochemical markers that, by comparison of their expression on preoperative biopsies and tumor downstaging (evaluated clinically or histologically on surgical specimens), may contribute to a better assessment of response and possibly provide additional prognostic information has emerged.

## 2. Methods

Here we provide a systematic analysis of the immunohistochemical markers most studied in the literature, trying to give a complete picture of the additional tools available to the pathologist that can give useful information about the response to therapy and prognosis to the oncologist.

### 2.1. Inclusion Criteria

Our review is focused on studies concerning immunohistochemical marker expression evaluated in patients with rectal cancer treated with neoadjuvant therapies (chemo- and/or radiotherapy) published in English during the last 10 years (i.e., 2006–2016). Other inclusion criteria consisted of studies that focused also on the prognostic value of each immunohistochemical marker identified, and included a minimum of 15 patients (Figure 1).

### 2.2. Search Terms

Studies were identified using the search engine PubMed (https://www.ncbi.nlm.nih.gov/pubmed). To identify pertinent studies, a set of search terms was elaborated. The final search parameter included the terms “immunohistochemistry,” “colorectal adenocarcinoma,” and “neoadjuvant.”

The full search code used is as follows, and it allowed us to identify 29 studies: (“immunohistochemistry”[MeSH Terms] OR “immunohistochemistry”[All Fields]) AND (colorectal[All Fields] AND (“adenocarcinoma”[MeSH Terms] OR “adenocarcinoma”[All Fields])) AND (“neoadjuvant therapy”[MeSH Terms] OR (“neoadjuvant”[All Fields] AND “therapy”[All Fields]) OR “neoadjuvant therapy”[All Fields] OR “neoadjuvant”[All Fields]).

### 2.3. Exclusion Criteria

Based on title and abstracts 11 studies were excluded, leaving 18 studies for further evaluation. Exclusion criteria were molecular biology studies and studies where the primary focus was the detection of marker expression by means other than immunohistochemistry.

## 3. Results

### 3.1. p53

p53 is an isoform of a protein encoded by a homologous gene, and it prevents cancer formation with a tumor-suppressing role.

p53 is involved in apoptosis, genomic stability, and inhibition of angiogenesis, acting through several mechanisms, such as DNA repair, arresting cell cycle at the G1/S regulation point, and activating apoptosis.

Approximately half of all colorectal cancers show p53 gene mutations, with higher frequencies observed in the distal colon and rectal tumors [5].

In rectal cancers treated with neoadjuvant therapies, no correlation has been identified between differences in p53 expression and local recurrence or survival [6].

Furthermore, in another work, overexpression of p53 and young age were proved to be independent variables associated with a good response to adjuvant therapy by stepwise linear regression [7].

Different results were found by Terzi et al., who did not find a correlation between expression of p53 with response to preoperative chemoradiotherapy and prognosis [8].

### 3.2. p27kip1

p27kip1 is a cell-cycle regulatory protein that interacts with CDK2 and CDK4, inhibiting cell cycle progression at G1 [9].

Recent studies have shown that decreased expression of p27kip1 is associated with high-grade tumors and a poor prognosis in colonic cancer [10].

An association between chemoradiotherapy and p27kip1 protein expression demonstrated how p27kip1 lower expression was correlated with a poor response to adjuvant treatment, without indications on local recurrence or survival [7].

In addition, the relationship between p27kip1 expression in pretreatment biopsy material and differences in survival was studied by Günther et al., concluding that this marker could not aid the targeting of treatment strategies in rectal cancer, and it cannot serve as a predictor of survival [11].

### 3.3. Thymidylate Synthase (TS), Thymidine Phosphorylase (TP), and Dihydropyrimidine Dehydrogenase (DPD)

These three metabolic enzymes of fluoropyrimidines are known to be reliable biomarkers that allow us to predict tumor response to 5-fluorurouracil-based chemotherapy in solid tumors.

*Thymidylate synthase* is an enzyme that plays a crucial role in de novo DNA synthesis.

Its inhibition leads to arrested cell proliferation, and lung tumors expressing high TS levels were shown to be resistant to chemotherapy [12].

*Thymidine phosphorylase*, also named platelet-derived endothelial cell growth factor, is an enzyme that leads to the activation of fluoropyrimidine, and it is a well-known angiogenic molecule [13, 14].

*Dihydropyrimidine dehydrogenase* is an enzyme involved in pyrimidine degradation, catalyzing the reduction of uracil and thymine. It has also a recognized role in the degradation of the chemotherapeutic drug 5-fluorouracil [15].

Lack of immunohistochemical expression of thymidylate synthase has been found to be associated with tumor down-staging after preoperative chemoradiotherapy but not after radiotherapy. No correlation was found between thymidylate synthase expression and local recurrence or survival in locally advanced rectal cancers treated with preoperative adjuvant therapies [6].

In addition, a significant association was seen between high TS expression in tumor or resection specimens and lack of response of the tumor to therapy. Finally, low TP expression in the resection specimens was significantly associated with nonresponse [16].

For DPD, no significant correlations are documented in the literature to date [16].

### 3.4. Survivin

Survivin is a small molecule involved in the regulation of cell cycle and inhibition of apoptosis, expressed during embryogenesis and silenced after birth, with a re-expression in 60 different human tumor lines used in the National Cancer Institute's cancer drug screening program [17].

Moreover, its re-expression is correlated with a more aggressive tumor phenotype and chemotherapy resistance [18].

In colonic cancer treated with chemoradiotherapy, controversial results are illustrated in the literature so far.

Terzi et al. did not find a correlation between expression of survivin with response to preoperative chemoradiotherapy and prognosis [8].

Kim et al., on the contrary, found that high survivin immunohistochemical expression in pretreatment tumor biopsy was associated with less tumor downstaging after preoperative chemoradiotherapy for locally advanced rectal cancer by evaluation on preoperative biopsies and using tumor downstaging as an end point [19].

### 3.5. Ki67 Proliferative Index

Ki67 proliferative index is a useful tool used by pathologists to estimate tumor cell proliferation.

Its prognostic role is widely accepted in a wide range of cancers, varying from breast to lung as well as neuroendocrine tumors [20–22].

There was no correlation between Ki67 proliferative index with response to preoperative chemoradiotherapy and prognosis [8].

### 3.6. CD133 and Cyclooxygenase 2

*CD133* is a membrane glycoprotein expressed in cancer stem cells [23], and recent results reveal its prognostic role in a spectrum of solid tumors such as lung [24], glioma [25], and ovarian [26] cancers.

*Cyclooxygenase 2* (COX2) is one of the two isoforms of COX involved in the metabolic conversion of arachidonic acid to prostaglandins, including prostaglandin E2 which is one of the main mediator of inflammation and angiogenesis [27, 28].

COX2 has been hypothesized to inhibit apoptosis, to promote angiogenesis and modulation of cell differentiation and even more to improve cancer aggressiveness and metastasizing potential [29].

Both these marker expressions were associated with chemoradioresistance in rectal cancer.

In detail, analyses of the degree of cytological alterations also revealed a significant association between chemoradioresistance and the expression of CD133 and cyclooxygenase 2 [30].

Another study did not find a predictive role in the treatment response of cyclooxygenase 2 in patients with rectal cancer treated with preoperative radiotherapy [31].

A study investigating CD133 mRNA expression in residual cancer cells after chemoradiotherapy (CRT) in rectal cancer showed how elevated post-CRT CD133 expression was associated with poor disease-free survival, and patients who developed distant recurrence had a higher post-CRT CD133 compared with those patients without recurrence. Immunohistochemically, CD133 was observed in residual cancer cells after CRT and its expression in residual cancer cells after CRT may indicate a treatment resistant phenotype [32].

### 3.7. Matrix Metalloproteinase-9

Matrix metalloproteinase-9 (MMP9) is a member of matrix metalloproteinases, a family of endopeptidases implied in degradation of extracellular matrix which leads to tissue repair and remodelling.

Overexpression of MMP9 has been found in various tumors, and recently an association with a poor prognosis in osteosarcoma and oral and gastric cancer has been proved [33–35].

Unsal Kilic et al. found that MMP9 expression correlated with a poor tumor response to preoperative chemoradiotherapy in rectal carcinoma patients [36].

### 3.8. CD44v6

CD44 glycoprotein is a member of cell adhesion molecules (CAMs) family, which mediates cell interactions by controlling cell-to-cell and cell-to-extracellular matrix contact and preserving tissue integrity.

Consequently, a dysregulation of their expression leads to increased tumor aggressiveness and distant metastasis.

V6 variant of CD44 has been proved to correlate with a bad prognosis in Hodgkin's lymphoma, colonic, cervical, gastric, and breast cancer [37].

Peng et al. found that CD44v6 expression in cancer cells was a sensitive marker for predicting the treatment outcome in patients with stage II and III adenocarcinoma of the rectum after total mesorectal excision and it could be used to determine whether an adjuvant treatment is necessary or not, but the authors concluded that further investigations are needed to determine the clinical application of CD44v6 and its reliability [38].

## 4. Discussion

To date, evaluation of response to medical therapy in colorectal cancer is based on clinical evaluation with posttreatment imaging and histologic examination with hematoxylin and eosin staining of the surgical specimen after surgery.

The different grading systems of tumor regression are based on pure morphology, and it could be helpful to identify a marker or panel of markers that could give more detailed information to the oncologist about response to therapy and survival.

To date, several markers have been studied and some of which described above gave controversial results in relation to rectal neoplasms.

In particular, p53 protein expression was not associated with neoadjuvant treatment response nor was it useful to predict local recurrence or survival.

p27 and MMP9 were associated just with the degree of response to chemoradiotherapy, without giving additional information about survival.

On the contrary, COX2 and CD133 expression was correlated with differences in survival in patients pretreated with chemoradiotherapy, but not with different histological responses to medical treatments.

Survivin expression studies gave controversial results, and just one study showed how this protein associated with neoadjuvant treatment response.

Studies about thymidine phosphorylase and thymidylate synthase concluded that the expression of these markers could be associated with response to just chemotherapy in rectal cancers, with no considerations about survival differences. In the same study, dihydropyrimidine dehydrogenase did not give useful information about response or survival.

CD44v6 expression in cancer cells was a sensitive marker for predicting the treatment outcome in patients with rectal adenocarcinoma, but the authors concluded that further investigations are needed to confirm these results.

Finally, there was no correlation between Ki67 proliferative index with response to preoperative chemoradiotherapy and prognosis.

In this view, further studies are needed to investigate newer immunohistochemical markers that could be associated with histomorphological examination and which may suggest new molecular targets also in order to elaborate targeted and patient-tailored therapies.

## Figures and Tables

**Figure 1:**